# Association of normal-weight central obesity with hypertension: a cross-sectional study from the China health and nutrition survey

**DOI:** 10.1186/s12872-023-03126-w

**Published:** 2023-03-08

**Authors:** Huihui Ren, Yaoyao Guo, Dan Wang, Xiaonan Kang, Gang Yuan

**Affiliations:** 1 Department of Endocrinology, Tongji Hospital, Tongji Medical College, Huazhong University of Science and Technology, Wuhan, 430030 Hubei People’s Republic of China; 2 Branch of National Clinical Research Center for Metabolic Disease, Hubei, People’s Republic of China

**Keywords:** Normal-weight central obesity, Central obesity, Body mass index, Hypertension, Cardiometabolic risk

## Abstract

**Background:**

Central obesity is associated with an increased risk of hypertension in the general population. However, little is known regarding the potential relationship between central obesity and the risk of hypertension among adults with a normal body mass index (BMI). Our aim was to assess the risk of hypertension among individuals with normal weight central obesity (NWCO) in a large Chinese population.

**Methods:**

We identified 10 719 individuals aged 18 years or older from the China Health and Nutrition Survey 2015. Hypertension was defined by blood pressure measurements, physician diagnosis, or the use of antihypertensive treatment. Multivariable logistic regression was used to assess the association of obesity patterns, defined by BMI, waist circumference (WC) and waist hip ratio (WHR), with hypertension after adjusting for confounding factors.

**Results:**

The patients’ mean age was 53.6 ± 14.5 years, and 54.2% were women. Compared with individuals with a normal BMI but no central obesity, subjects with NWCO had a greater risk of hypertension (WC: OR, 1.49, 95% CI 1.14–1.95; WHR: OR, 1.33, 95% CI 1.08–1.65). Overweight-obese subjects with central obesity demonstrated the highest risk of hypertension after adjustment for potential confounders (WC: OR, 3.01, 95% CI 2.59–3.49; WHR: OR, 3.08, CI 2.6–3.65). Subgroup analyses showed that the combination of BMI with WC had similar findings to the overall population except for female and nonsmoking persons; when BMI was combined with WHR, a significant association of NWCO with hypertension was observed only in younger persons and nondrinkers.

**Conclusions:**

Central obesity, as defined by WC or WHR, is associated with an increased risk of hypertension in Chinese adults with normal BMI, highlighting the need to combine measures in obesity-related risk assessment.

## Introduction

The prevalence of obesity has increased rapidly in recent years, and it has become a major challenge for health policy-makers [1, 2]. The global epidemic of obesity has led to substantial health and economic costs and affected more than 2 billion people, including more than 11% of men and 15% of women [2]. In China, an alarming increase in overweight and obesity has been observed in the past four decades, and in 2015–2019, the estimated national prevalence for overweight and obesity was 34.3% and 16.4%, respectively [3]. Obesity is a leading risk factor for noncommunicable disease morbidity and mortality. Obesity is often associated with a higher likelihood of finding altered metabolic and cardiovascular risk factors, such as hypertension, diabetes, dyslipidemia and stroke [4]. The rise in obesity frequently leads to premature death and future falls in life expectancy in the general population [5, 6]. The epidemic of obesity and its devastating threat to health have posed an enormous public health burden and overwhelming financial burden on health care systems [7, 8].

Despite the fact that body mass index (BMI) has been widely used for identifying obesity in clinical practice, a significant limitation of BMI is that it does not distinguish between different body shapes or body compositions [9]. An individual with normal weight may have an increased body fat percentage that might be masked by their normal BMI value. Existing evidence has suggested that central obesity, characterized by relatively high abdominal fat distribution, is more strongly associated with cardiometabolic risk factors than general obesity [10, 11]. Even among individuals with normal weight, those with a high body fat percentage have a higher prevalence of metabolic syndrome and its components than those with a normal BMI and a normal body fat percentage [12]. Recently, multiple studies have examined the association of central obesity with the risk of cardiometabolic disease and mortality in the general population [13–15]. However, it remains unclear whether the association is maintained in adults with normal BMI. Normal-weight central obesity (NWCO) is a metabolic condition that has been recently described in a few studies [16, 17]; individuals with NWCO are easily ignored in routine health care due the strict focus on the BMI.

Hypertension is a very common complaint in the general population and leads to an enormous social burden and growing mortality around the world [18]. According to the World Health Organization, an estimated 1.28 billion adults aged 30–79 years worldwide will suffer from hypertension in 2021 [19]**.** The burden of hypertension in China is increasing along with urbanization, rising incomes, and aging of the population, reaching 44.7% among Chinese adults aged 35–75 years in 2017 [20]. The epidemic of hypertension undoubtedly imposes a challenging burden for a clustering of cardio-metabolic disorders, including cardiovascular disease (CVD), chronic kidney disease, and mortality [21–23]. Studies have indicated that the rise in hypertension prevalence occurs in parallel with lifestyle changes and obesity epidemics. Findings from previous studies suggested that both general and central obesity are associated with a greater risk of hypertension [24, 25]. Hence, it is of great importance to control weight and adjust lifestyle factors to prevent the onset of hypertension and subsequent adverse health risks [26, 27]. However, little is known about whether the combination of BMI with measures of central obesity, such as waist circumference (WC) and waist hip ratio (WHR), enables better discrimination of participants at greater risk of hypertension and how subjects with NWCO fare in comparison to subjects with other body adiposity patterns.

To our knowledge, no studies in the Chinese population have specifically focused on assessing the risk of hypertension in persons with NWCO. We aimed to determine whether NWCO confers greater risk of hypertension compared with subjects with normal, overweight and obese BMI who do not have central obesity. We took advantage of the data from the China Health and Nutrition Survey (CHNS) to investigate the risk of hypertensions associated with different patterns of body adiposity based on a combination of BMI and either WC or WHR.

## Materials and methods

### Study design and participants

The CHNS is an ongoing nationwide survey that was designed to examine the health and nutritional status of the Chinese population. The survey started in 1989 and was followed up every 2–4 years for a total of 10 waves. The available data of the CHNS survey are currently updated to 2015. Details of the survey have been published elsewhere [28]. A stratified multistage, random-cluster design method was used to obtain nationally representative samples from 12 provinces that vary in geography, economic development, and health status. Counties and cities within the provinces were stratified by income (low, middle and high), and a weighted sampling scheme was used to randomly select four counties and two cities in each province. Finally, 20 households in each community were randomly selected, and all household members were interviewed. The data were collected by a team of interviewers who received intensive training before they could start working. Interviewers should make a house visit to collect the information using a structured questionnaire. The study protocol for the survey was approved by the institutional review board of the University of North Carolina at Chapel Hill, the National Institute for Nutrition and Health, and the Chinese Center for Disease Control and Prevention. Written informed consent was obtained from all subjects.

**Fig. 1:**
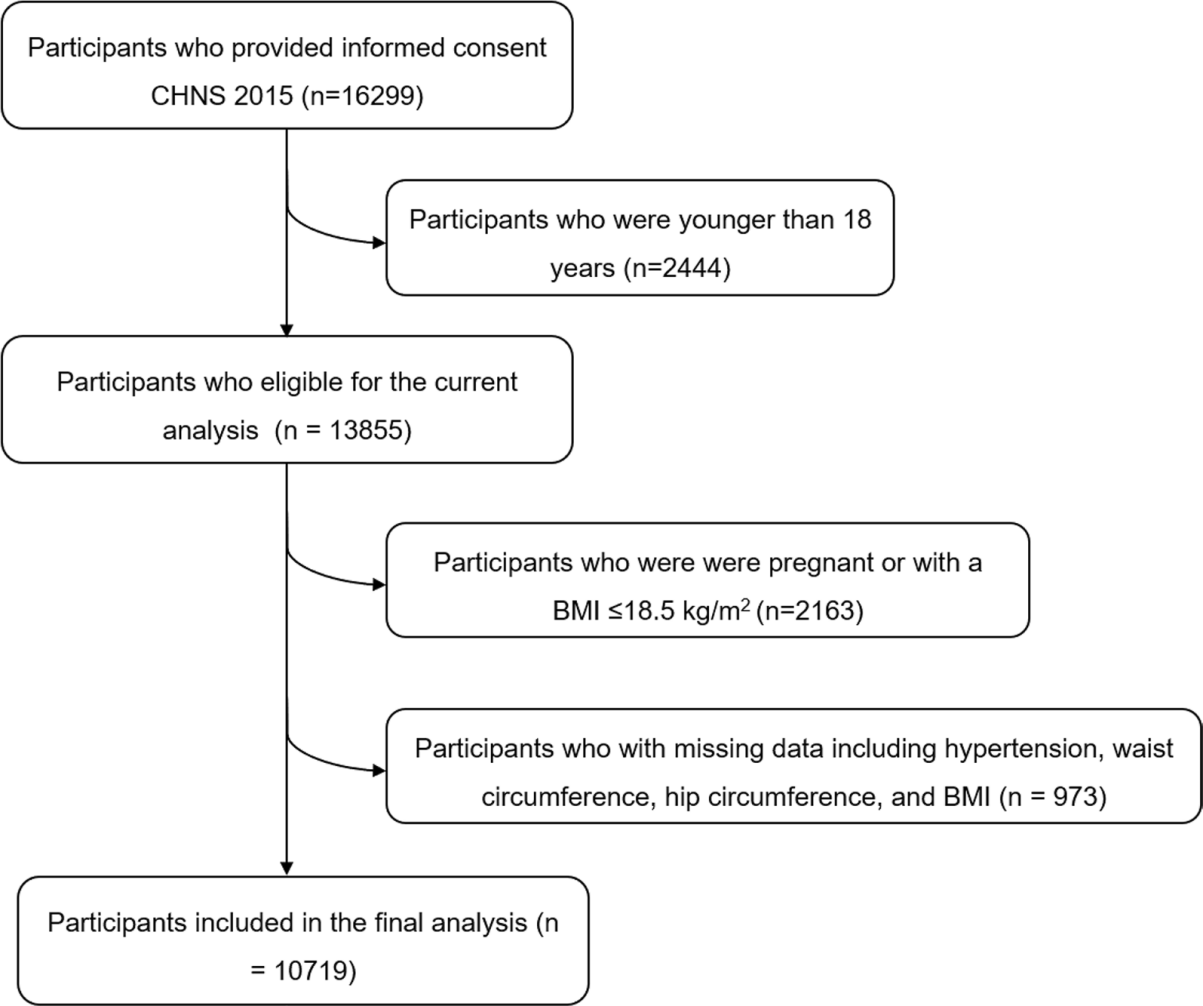
Flowchart for patient’s selection

A total of 13,855 individuals eligible for the current analysis were aged 18 years or older and participated in the 2015 CHNS survey. We excluded 2163 participants who were pregnant or had a BMI ≤ 18.5 kg/m^2^. Furthermore, participants with missing data, including hypertension, WC, hip circumference, and BMI, were also excluded. The final participants in this study included 10,719 participants (4914 men and 5805 women). A flowchart is shown in Fig. 1.

### General and central obesity categories

The weight of individuals was estimated to the nearest 0.1 kg with light clothing using a calibrated beam scale (Seca North America, Chino, CA, USA). Height was measured with the participants barefoot to the nearest 0.1 cm using a portable stadiometer (Seca North America). BMI was calculated by the following formula: weight (kg) divided by height (m^2^). The study used the Working Group on Obesity in China recommendations for general obesity [29]. Obesity was defined as BMI ≥ 28 kg/m^2^, overweight as a BMI of 24–27.9 kg/m^2^, and normal BMI as a BMI of 18.5–24.9 kg/m^2^. WC was measured midway between the lower rib and iliac crest, while hip circumference was measured at the level of the major trochanter. The WHR was calculated as the ratio of waist-to-hip circumference, both in centimeters. High WC was defined as 85 cm or more for women and 90 cm or more for men according to the Chinese-specific abdominal obesity standard [30]. High WHR was defined as 0.85 or more for women and 0.90 or more for men [31]. Participants were defined as having central obesity if they had either the WHR or the WC above the sex-specific cutoff point.

### Measurements and definition of hypertension

In the survey, the history of hypertension of participants was collected via questionnaires through the question ‘Has a doctor ever told you that you suffer from high blood pressure?’. Those who answered ‘yes’ were further asked “Are you currently taking anti-hypertension drugs?”. Regularly calibrated mercury sphygmomanometers (subdivision:

2 mmHg, measuring scope: 0–300 mmHg) were used to measure blood pressure by trained examiners. Measurements were performed in triplicate after at least a 10-min rest, and the mean of three measurements was considered the corresponding value to reduce measurement errors. The definition of hypertension was confirmed in the presence of one or more of the three components: (1) systolic/diastolic blood pressure (SBP/DBP) ≥ 140/90 mmHg, (2) a previous diagnosis of hypertension, and (3) current use of anti-hypertension agents.

### Confounding variables and definitions

Data including demographics, personal history, and unhealthy habits were collected using a standardized questionnaire. Sociodemographic information included age, sex (men, women), setting (urban, rural), marital status (married, never married, separated/divorced/widowed), and education level (no education/primary school/secondary school, high school and above). Lifestyle characteristics included smoking (never/former, current smokers), drinking, TV time and sleep duration. Alcohol drinking status in the last year was assessed and categorized as either ‘ever’ or ‘never’. Sleep duration was based on participant self-report. The question asked was: How many hours each day do you usually sleep, including daytime and nighttime? (hours). The variable on time spent sleeping was used as a continuous variable (hours/day) and as a categorical variable (< 7, 7–8,  ≥ 9 h/day) according to our previous study [32]. Participants reported total time spent watching TV in the previous week. The total time spent watching TV per week was used to create three categories of TV viewing based on the cutoff value as described in previous publications [33]: (0–7, 7.01–14 and > 14 h per week).

### Statistical analysis

To examine the effect of each anthropometric category on risk of hypertension, obesity patterns were categorized into 8 groups on the basis of the combination of BMI and central obesity category: (1) normal BMI/low WHR, (2) normal BMI/high WHR, (3) overweight/obese BMI/low WHR, (4) overweight/obese BMI/high WHR, (5) normal BMI/low WC, (6) normal BMI/high WC, (7) overweight/obese BMI/low WC, and (8) overweight/obese BMI/high WC. Baseline characteristics were presented according to gender and the combinations of BMI and WC categories. Continuous variables are expressed as the mean ± SD, while categorical variables are presented as numbers (%). We compared the characteristics of the study population using Student’s* t* test or variance for continuous variables and *χ*2 tests for categorical variables.

Univariate and multivariable-adjusted logistic regression models were used to investigate the association of these anthropometric categories with hypertension. The results are presented as odds ratios (ORs) and 95% confidence intervals (CIs) for categories of these anthropometric variables. Three models were conducted. Model 1 presented the crude ORs and 95% CIs for the risk of hypertension. Model 2 was adjusted for sociodemographic variables (age, sex, education, marital status, setting), and model 3 was further adjusted for potential factors, including smoking status, alcohol consumption, sleep duration, and TV time.

We performed a stratified analysis to evaluate whether the associations varied by age (< 64 vs. ≥ 65 years), sex (men, women), smoking status (current vs. ever/never smoker) and alcohol consumption (yes vs. no). Potential interactions between obesity patterns and these stratifying variables were assessed by adding cross-product terms to the multivariable regression models. The statistical analyses were performed using SPSS software version 19.0 (Chicago, Illinois, USA). The level of statistical significance was set at a *P* value of less than 0.05.

## Results

The mean age of the 10,719 survey participants in the analysis was 53.6 ± 14.5 years, and 5805 (54.2%) were women. Baseline characteristics of participants stratified by sex are presented in Table 1. Male participants were more likely to be married, current smokers, consume more alcohol and have a higher education and longer sleep duration (*P* < 0.05). Additionally, men had higher median values for anthropometrics, including height, weight, HC, WC, and WHR, than women (*P* < 0.05). No significant differences were observed in the distribution of age, setting, TV time, BMI or medical history of hypertension between sexes.

**Table 1:** Baseline characteristics of study participants stratified by sex*

Variable	Men	Women	*P* value
	*n* = 4914	*n* = 5805	
Age (years)	53.8 ± 14.5	53.4 ± 14.5	0.110
Education, < high school graduation	2473 (55.9)	2733 (60.5)	0.001
Setting, urban, *n* (%)	1909 (38.8)	2289 (39.4)	0.538
Marital status, married, n (%)	4413 (90)	4967(85.8)	0.001
Smoking, current, *n* (%)	2602 (53.1)	134(2.3)	0.001
Drinking, last year alcohol, *n* (%)	2588 (52.7)	325(5.6)	0.001
Hypertension, *n* (%)	893 (18.2)	1071(17.5)	0.389
TV time (hour/week)	17.7 ± 16	17.2 ± 15.1	0.051
Sleep duration(hour)	7.8 ± 1.2	7.7 ± 1.3	0.039
*Anthropometry*			
Weight (kg)	68.9 ± 11.5	59.9 ± 9.7	0.001
Height (cm)	167.2 ± 7.1	156.1 ± 6.8	0.001
HC (cm)	96.0 ± 10.3	95.2 ± 10.6	0.001
WC (cm)	87.1 ± 12.4	83.2 ± 12.8	0.001
WHR	0.92 ± 0.4	0.89 ± 0.5	0.001
BMI (kg/m^2^)	24.6 ± 3.8	24.6 ± 4.0	0.566

*WC* Waist circumference, *HC* Hip circumference, *WHR* Waist-to-hip ratio, *BMI* Body mass index

^*^Values are mean ± SD or No. (percentage) unless otherwise indicated

According to the BMI of the survey participants, 5034 (47.0%) were normal (18.5 to 23.9 kg/m^2^), 4034 (37.6%) were overweight (24 to 279 kg/m^2^), and 1651 (25.1%) were obese (≥ 28.0 kg/m^2^). Among those with normal BMI, 672 participants (13.3%) were categorized as centrally obese using a sex-specific cutoff value for WC. Of those who were overweight-obese according to BMI, 3698 adults (65.0%) had a large WC. Table 2 shows the characteristics of the survey participants according to the BMI/WC combination. In any given BMI category, individuals with central obesity were more likely to be older, female, have less sleep time, and have a high rate of divorce or being single than those without central obesity (*P* < 0.05). Notably, there were more patients with hypertension in the normal weight/high WC (19.5%) category than in the normal weight/normal WC (10.4%) and overweight-obese/normal WC (16.6%) categories (*P* < 0.05). In the normal BMI category, adults with central obesity were more likely to be urban and less likely to smoke and drink alcohol than men with similar BMI but no central obesity (*P* < 0.05). However, no significant difference was observed in these variables between the central obesity and noncentral obesity groups in the overweight-obese category (*P* > 0.05). In addition, there was no difference in education level or TV time between the central obesity and noncentral obesity groups across BMI categories (*P* > 0.05).

**Table 2:** Baseline characteristics of the survey participants according to the BMI and WC combinations*

Variable	Normal Weight			Overweight -obesity		
	Normal WC	High WC	*P* value	Normal WC	High WC	*P* value
Participants	4362	672		1987	3698	
Age (years)	52 ± 15.3	58.1 ± 15	0.0001	52 ± 13.7	55.5 ± 13.3	0.001
Male, *n* (%)	2015 (46.2)	23.1 (34.4)	0.0001	990 (49.8)	1678 (45.4)	0.001
Education, < high school graduation	2079 (57)	311 (58.2)	0.607	985 (57.5)	1831 (60.1)	0.080
Setting, urban, n (%)	1617 (37.1)	315 (46.7)	0.0001	780 (39.3)	1486 (40.2)	0.514
*Marital status, n* (%)						
Married	3775 (86.7)	575 (86.1)	0.0001	1780 (89.9)	3250 (88.1)	0.001
Never married	247 (5.7)	19 (2.8)		70 (3.5)	86 (2.3)	
Separated/divorced/widowed	332 (7.6)	74 (11.1)		129 (6.5)	355 (9.6)	
Smoking, current, *n* (%)	1183 (27.2)	134 (20)	0.0001	507 (25.6)	912(24.7)	0.479
Dinking, last year alcohol, *n* (%)	1171 (26.9)	141 (21)	0.001	573 (28.9)	1028 (27.8)	0.386
Hypertension, *n* (%)	455 (10.4)	131 (19.5)	0.0001	330 (16.6)	994 (26.9)	0.001
*TV time (hour/week)*						
0–7	1487 (34.3)	209 (31.4)	0.349	663 (33.6)	1130 (30.7)	0.076
7.01–14	1387 (32)	220 (33.1)		636 (32.3)	1255 (34.1)	
> 14 h	1465 (33.8)	236 (35.5)		672 (34.1)	1293 (35.2)	
*Sleep duration(hour)*						
< 7	508 (11.7)	101(15.3)	0.0001	205 (10.4)	495 (13.5)	0.001
7–8	3048 (70.4)	410(61.9)		1455 (74.1)	2525 (69.1)	
≥ 9	773 (17.9)	151(22.8)		303 (15.4)	636 (17.4)	
*Anthropometry*						
Weight (kg)	56.1 ± 6.9	58.7 ± 7.7	0.0001	66.1 ± 8.1	73.3 ± 10.6	0.001
Height (cm)	160.9 ± 8.2	161.6 ± 9.3	0.059	159.9 ± 9.3	162.1 ± 9.2	0.001
HC (cm)	90.7 ± 7.8	95.4 ± 8.9	0.0001	93.9 ± 12.3	102.3 ± 8.6	0.001
WC (cm)	76.5 ± 9.7	92.3 ± 5	0.0001	80.7 ± 12.6	95.9 ± 6.9	0.001
WHR	0.86 ± 0.44	0.98 ± 0.11	0.0001	0.89 ± 0.46	0.96 ± 0.43	0.001
BMI (kg/m^2^)	21.6 ± 1.5	22.4 ± 1.3	0.0001	25.8 ± 3.4	27.9 ± 3.6	0.001

*WC* Waist circumference, *HC* Hip circumference, *WHR* Waist-to-hip ratio, *BMI* Body mass index

^*^Values are mean ± SD or No.(percentage) unless otherwise indicated. Normal weight, overweight, and obese were defined using standard BMI cutoffs (normal BMI, 18.5–23.9 kg/m2; overweight, 24–27.9 kg/m2; obese, ≥ 28 kg/m2). High WC was defined as > 85 for women and > 90 for men

The multivariate logistic model results for different combinations of BMI, central adiposity, and hypertension are presented in Table 3. Using normal BMI/low WC as the reference group, the crude ORs (95% CIs) for the association between normal BMI/high WC and hypertension were 2.08 (95% CI 1.68–2.58) and 1.71 (95% CI 1.47–2.0) for the association between overweight-obesity/low WC and hypertension. The overweight-obese/high WC group had the highest risk of hypertension (OR, 3.16; 95% CI 2.8–3.56). When controlling for potential cofounders, the association of normal BMI/high WC with hypertension was decreased (OR 1.49, 95% CI 1.14–1.95), and there was no significant change in the other two groups. Using normal BMI/low WHR as the reference group, the risk of hypertension increased gradually across the BMI/WHR categories (normal BMI/high WHR: OR, 1.7, 95% CI 1.43–2.02; overweight-obese/low WHR: OR 1.83, 95% CI 1.52–2.19; overweight-obese/high WHR: OR 3.39, 95% CI 2.95–3.9). The association between BMI/WHR and hypertension was still maintained after additional adjustment for potential confounders.

**Table 3:** Hypertension Risk-Combination of BMI, WC, and WHR and Independent Anthropometric Measures of Obesity

	Model1	Model2	Model3
	OR (95% CI)	OR (95% CI)	OR (95% CI)
*Combination of BMI and WC*			
Normal BMI/low WC	Reference	Reference	Reference
Normal BMI/high WC	2.08 (1.68–2.58)	1.45 (1.11–1.89)	1.49 (1.14–1.95)
Overweight-obese/low WC	1.71 (1.47–2)	1.87 (1.55–2.24)	1.85 (1.53–2.22)
Overweight-obese/high WC	3.16 (2.8–3.56)	3 (2.58–3.46)	3.01 (2.59–3.49)
*Combination of BMI and WHR*			
Normal BMI/low WHR	Reference	Reference	Reference
Normal BMI/high WHR	1.7 (1.43–2.02)	1.31 (1.06–1.61)	1.33 (1.08–1.65)
Overweight-obese/low WHR	1.83 (1.52–2.19)	1.91 (1.54–2.38)	1.89 (1.52–2.36)
Overweight-obese/high WHR	3.39 (2.95–3.9)	3.06 (2.59–3.62)	3.08 (2.6–3.65)
*Independent anthropometric measures*			
Normal BMI	Reference	Reference	Reference
Overweight-obese BMI	1.98 (1.77–2.23)	2.01 (1.75–2.31)	2.01 (1.74–2.31)
Obese BMI	3.19 (2.78–3.65)	3.75 (3.17–4.44)	3.74 (3.16–4.43)
Low WC	Reference	Reference	Reference
High WC	2.46 (2.22–2.72)	2.16 (1.91–2.44)	2.18 (1.93–2.47)
Low WHR	Reference	Reference	Reference
High WHR	2.2 (1.97–2.45)	1.86 (1.64–2.12)	1.9 (1.66–2.16)

*WC* Waist circumference, *HC* Hip circumference, WHR Waist-to-hip ratio, *BMI* Body mass index

Model 1 showed a crude model. Model 2 were adjusted for age, sex, education, marital status, and setting. Model 3 further adjusted for smoking status, alcohol consumption, sleep duration, and TV time

^*^Values are mean ± SD or No. (percentage) unless otherwise indicated. Normal weight, overweight, and obese were defined using standard BMI cutoffs (normal BMI, 18.5–23.9 kg/m2; overweight, 24–27.9 kg/m2; obese, ≥ 28 kg/m2). High WC was defined as > 85 for women and > 90 for men. High WHR was defined as > 0.85 for women and > 0.90 for men.

On the basis of BMI category alone, there was an increase in the risk of hypertension as the BMI category increased from normal weight to overweight to obese. Upon evaluation based on WC alone, patients with high WC were more than 2.0 times more likely to have hypertension (OR, 2.18; 95% CI 1.93–2.47) than patients with normal WC. A high WHR alone was associated with a 90% higher risk of hypertension (OR, 1.90; 95% CI 1.66–2.16) than a normal WHR.

**Fig. 2:**
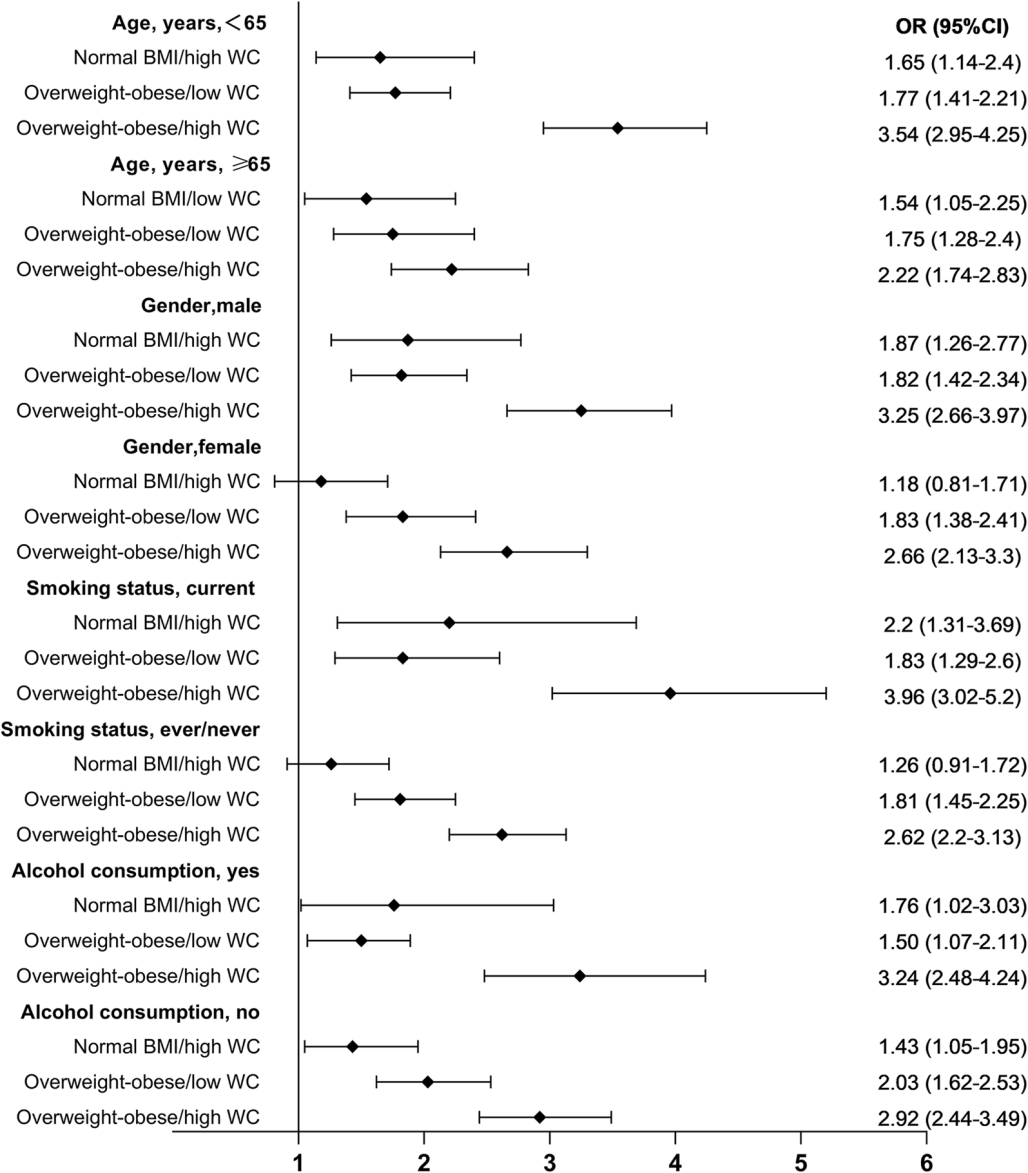
Stratified analyses for the association of hypertension with the combinations of BMI and WC. Normal weight, overweight, and obese were defined using standard BMI cutoffs (normal BMI, 18.5–23.9 kg/m2; overweight, 24–27.9 kg/m2; obese,  ≥ 28 kg/m2). High WC was defined as > 85 for women and > 90 for men. High WHR was defined as > 0.85 for women and > 0.90 for men. All values were OR (95% CI). Models were adjusted for age, sex, education, marital status, setting, smoking status, alcohol consumption, sleep duration, and TV time, except for the stratifying factor

**Fig. 3:**
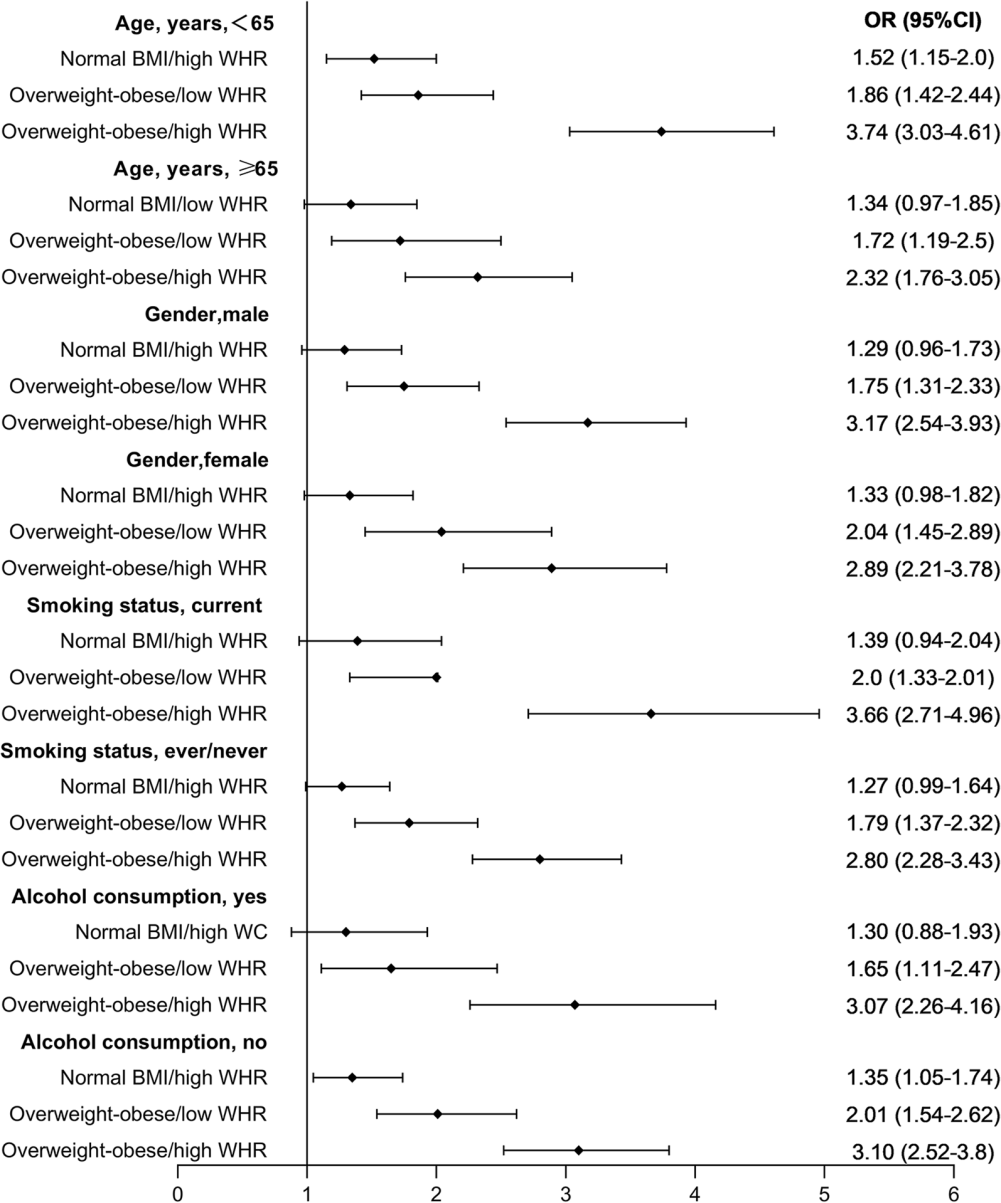
Stratified analyses for the association of hypertension with the combinations of BMI and WHR. Normal weight, overweight, and obese were defined using standard BMI cutoffs (normal BMI, 18.5–23.9 kg/m2; overweight, 24–27.9 kg/m2; obese, ≥ 28 kg/m2). High WC was defined as > 85 for women and > 90 for men. High WHR was defined as > 0.85 for women and > 0.90 for men. All values were OR (95% CI). Models were adjusted for age, sex, education, marital status, setting, smoking status, alcohol consumption, sleep duration, and TV time, except for the stratifying factor

Stratified analyses for the association of hypertension with different combinations of BMI and WC/WHR are represented in Fig. 2 and Fig. 3. In the combination of BMI and WC, the effect of central obesity on risk of hypertension was pronounced in all of the subgroups, except for the subgroup with female and nonsmoking participants (OR, 1.18, 95% CI 0.81–1.71; OR, 1.26, 95% CI 0.91–1.72, respectively). Of note, individuals with normal-weight central obesity had a higher risk of hypertension than those who were overweight-obese but had no central obesity in the male, smoking and drinking subgroups. In the combination of BMI and WHR, we only observed a substantial relationship of normal weight central obesity with hypertension among those who were younger and nondrinkers (OR, 1.52, 95% CI 1.15–2.0; OR, 1.35, 95% CI 1.051–1.74, respectively). The interactions showed no significant difference (*P* for interaction > 0.05). These results indicated that WC was a more valuable predictor of hypertension than WHR in the subgroup population. Using normal BMI/low WC or BMI/low WHR as the reference group, the risk of hypertension was higher among overweight and obese individuals in the presence or absence of central obesity.

## Discussion

Our analyses of data from a large cohort of CHNS participants showed that even in persons with normal weight according to BMI, measures of central obesity, such as WC and WHR, were independently associated with an increased risk for hypertension after adjustment for potential confounders. When combined with BMI, WC and WHR may provide additional prognostic value. Additionally, we suggested that a combination of BMI and WC compared with WHR provided an improved risk stratification of sex, age, drinking and smoking. Our findings support the concept that clinicians should go beyond BMI when assessing cardiometabolic risk in individuals with normal weight because combining BMI with measures of central obesity allows us to better discriminate those at the highest and lowest risk.

An obesity epidemic is occurring in China, which is related to health risks. The cardiometabolic effects of obesity on insulin sensitivity, inflammation, oxidative states, and subsequent increased CVD and mortality risk have been a frequent target of scientific research. Although BMI is a popular anthropometric index for the measurement of obesity across various populations, it does not account for body fat distribution [17]. Compared with BMI, measures of abdominal adiposity, such as WC and WHR, appeared to be more effective indices to identify cardiometabolic risk factors and mortality. Hypertension prevalence is increasing with respect to weight gain and has major consequences on human health [34]. In the present study, the prevalence of hypertension in subjects with NWCO was obviously higher than that in subjects with normal BMI but no central obesity but equal to or greater than that in overweight-obese subjects without central obesity. Previous studies have explored the associations of BMI and central obesity, either separately or in combination, with risk of hypertension; however, very few studies have evaluated the risk of hypertension among people with NWCO. The results in our study demonstrated that having abdominal obesity, measured by WC or WHR, even in persons with normal BMI, will also increase the risk of developing hypertension. To our knowledge, our study is the largest study to investigate the association of NWCO with hypertension. Our findings have significant clinical implications because adults with NWCO are not considered a priority population for prevention programs by guideline developers.

Our study not only specifically evaluated the risk of hypertension associated with central obesity in persons with normal weight but also addressed the risk with BMI-defined overweight-obese persons with or without central obesity. For predicting hypertension, the OR of NWCO was significantly greater than that of similar BMI but no central obesity. In addition, the ORs gradually increased from the normal group to the NWCO, overweight-obese but no central obesity, and overweight-obese with central obesity groups. Our findings suggested that subjects with overweight-obese central obesity have a higher risk of hypertension than subjects with any other combination of BMI with WC or WHR. Studies have argued that BMI alone is unsatisfactory for predicting the cardiometabolic risk and mortality associated with obesity [35, 36]. A recent longitudinal study using data from the Korean National Health Insurance Service database demonstrated a discrepancy in the risk for major adverse cardiovascular events between general and abdominal obesity [37]. Coutinho et al. [38] indicated that measures of fat distribution, such as WC and WHR, are directly associated with mortality among subjects with coronary artery disease (CAD) in contrast to BMI. In addition, they have also demonstrated that normal weight with central obesity (normal BMI but high WHR) was associated with the highest risk of mortality in people with CAD [39]. In line with our results, these data suggested that at any BMI level, an increased proportion of abdominal fat as determined by an elevated WC or WHR was associated with increased cardiometabolic risk, indicating that it is worth exploring an effective and simple index of abdominal obesity beyond BMI in individual health risk assessments.

Several mechanisms support the effect of abdominal obesity on increasing blood pressure. Excessive fat accumulation in adipose tissue and ectopic sites results in impaired adipogenesis, adipokine dysregulation, increased proatherogenic inflammatory factors, circulating free fatty acids, oxidative stress, and lipotoxicity, leading to atherosclerosis and endothelial cell dysfunction and ultimately cardiometabolic disease through modulation of risk factors such as hypertension, diabetes mellitus, dyslipidemia, and metabolic syndrome [40]. Studies have also suggested mechanisms to explain why measures of central obesity are superior to BMI in predicting cardiometabolic risk. Individuals demonstrated remarkable variation in body fat distribution for a given BMI. Although BMI has been associated with higher cardiovascular risk factors, not every subject who is overweight or obese exhibits alterations in cardiovascular risk factors that are expected from a greater burden of body fat [35]. In a large international cardiometabolic CT imaging study, it was reported that within each specific BMI unit, an elevated WC was predictive of an increased accumulation of visceral adipose tissue [10]. NWCO subjects tend to develop a characteristic metabolic profile associated with excess body fat, such as a low-grade proinflammatory state, higher oxidative stress, insulin resistance, and lipid abnormalities, which result in increased risks of metabolic syndrome and cardiovascular disease.

One of the strengths of the current study is the large sample size from a national population from China. Additionally, our current study provides comprehensive evidence of different.

Obesity patterns defined by BMI, WC and WHR and their association with hypertension among a large Chinese population where there was no such evidence previously. Nevertheless, our study has several limitations that should be considered. First, it is a cross-sectional study that investigates associations but cannot provide evidence of causality. Second, we did not distinguish between overweight and obese states in evaluating BMI because of the insufficient sample size in the analysis. Third, there might be limitations regarding the quality of anthropometric measurements. Although the measurements were all completed by the same trained group, the anthropometric measurements were not repeated in a subsample of the multicentric and populational studies. Finally, we were unable to analyze the most common biochemical indicators in our study, as the data were unavailable from the CHNS database.

## Conclusions

In summary, the present study indicated that measures of abdominal obesity could be an effective screening index for hypertension among nonobese Chinese individuals. Furthermore, WC could be used to assess individual risk of hypertension irrespective of age, sex, smoking habits, alcohol consumption, and sedentary behavior. WC has better discriminatory power than WHR in subgroups of individuals.

## Data Availability

The China Health and Nutrition Survey data are publicly available from https://www.cpc.unc.edu/projects/China.

## Abbreviations

BMI: Body mass index
NWCO: Normal-weight central obesity
WC: Waist circumference
WHR: Waist-hip ratio
CVD: Cardiovascular disease
OR: Odds ratio
CI: Confidence interval
CHNS: China health and nutrition survey
HC: Hip circumference
CAD: Coronary artery disease
